# Oncogenic activity of poly (ADP-ribose) glycohydrolase

**DOI:** 10.1038/s41388-018-0568-6

**Published:** 2018-11-20

**Authors:** Maud Marques, Maika Jangal, Li-Chun Wang, Anna Kazanets, Sabrina Daniela da Silva, Tiejun Zhao, Amanda Lovato, Henry Yu, Su Jie, Sonia del Rincon, John Mackey, Sambasivarao Damaraju, Moulay Alaoui-Jamali, Michael Witcher

**Affiliations:** 1 Departments of Oncology and Experimental Medicine, The Lady Davis Institute of the Jewish General Hospital, McGill University, Montreal, QC Canada; 2 Department of Otolaryngology/Head and Neck Surgery, Sir Mortimer B. Davis-Jewish General Hospital, McGill University, Montreal, QC Canada; 3 Department of Oncology, Division of Medical Oncology, Cross Cancer Institute, University of Alberta, Edmonton, AB Canada; 4 Department of Laboratory Medicine and Pathology, Cross Cancer Institute, University of Alberta, Edmonton, AB Canada

**Keywords:** Oncogenes, Breast cancer, PolyADP-ribosylation

## Abstract

Poly (ADP-ribosylation), known as PARylation, is a post-translational modification catalyzed by poly (ADP-ribose) polymerases (PARP) and primarily removed by the enzyme poly (ADP-ribose) glycohydrolase (PARG). While the aberrant removal of post-translation modifications including phosphorylation and methylation has known tumorigenic effects, deregulation of PARylation has not been widely studied. Increased hydrolysis of PARylation chains facilitates cancer growth through enhancing estrogen receptor (ER)-driven proliferation, but oncogenic transformation has not been linked to increased PARG expression. In this study, we find that elevated PARG levels are associated with a poor prognosis in breast cancers, especially in HER2-positive and triple-negative subtypes. Using both in vitro and in vivo models, we demonstrate that heightened expression of catalytically active PARG facilitates cell transformation and invasion of normal mammary epithelial cells. Catalytically inactive PARG mutants did not recapitulate these phenotypes. Consistent with clinical data showing elevated PARG predicts poor outcomes in HER2+ patients, we observed that PARG acts in synergy with HER2 to promote neoplastic growth of immortalized mammary cells. In contrast, PARG depletion significantly impairs the growth and metastasis of triple-negative breast tumors. Mechanistically, we find that PARG interacts with SMAD2/3 and significantly decreases their PARylation in non-transformed cells, leading to enhanced expression of SMAD target genes. Further linking SMAD-mediated transcription to the oncogenicity of PARG, we show that PARG-mediated anchorage-independent growth and invasion are dependent, at least in part, on SMAD expression. Overall, our study underscores the oncogenic impact of aberrant protein PARylation and highlights the therapeutic potential of PARG inhibition in breast cancer.

## Introduction

Breast cancer is the most common cancer afflicting the female population, representing 29% of all newly diagnosed cases and is the second leading cause of death in the same population [1]. Based on the expression status of the estrogen receptor (ER), progesterone receptor, the human epidermal growth factor receptor 2 (HER2) and the proliferation marker Ki-67, breast cancer can be classified into five clinically relevant, pathological subtypes: luminal A, luminal B, triple-negative/basal-like, HER2-enriched and normal-like [2, 3]. Primary tumors are generally well managed with a combination of neoadjuvant chemotherapy, surgery and radiotherapy. Nevertheless, even if a minority of women are diagnosed with stage VI disease, nearly 30% of women diagnosed with early-stage disease will develop metastatic lesions over time [4]. Similar to other cancers, approximately 90% of breast cancer mortalities result from such metastasis [5]. Clearly, more effective therapeutic approaches are needed. One therapeutic avenue being widely explored is the targeting of enzymes involved in protein post-translational modification.

Post-translational modification of cellular proteins is a tightly regulated process that is commonly disturbed across cancer types providing cells with survival, proliferative and metastatic advantages. Both “writers” of protein modification and “erasers” may be aberrantly activated or overexpressed provoking widespread changes to signaling pathways and gene expression. While “writers”, such as tyrosine kinases, have been more thoroughly studied, it is also clear that “erasers”, such as histone deacetylases [6], phosphatases [7] and lysine demethylases [8], are overexpressed in tumors contributing to oncogenic phenotypes and representing druggable targets.

Poly (ADP)-ribosylation (referred to as PARylation) is a post-translational modification mediated primarily by poly (ADP-ribose) polymerases 1/2 (PARP1/2) in the nucleus and is reversible through the actions of poly (ADP-ribose) glycohydrolase (PARG) [9, 10]. Targeting the poly (ADP-ribose) pathway holds great promise as an approach to anti-cancer therapy, and some success has been met using PARP1/2 inhibitors (PARPiS) to treat cancers harboring defects in the homologous recombination repair pathway, especially BRCA1/2 mutated tumors. However, the use of PARPiS in clinical trials targeting tumors beyond these cohorts has been met with only moderate success [11, 12].

Beyond a role in DNA damage repair, PARP1/2 is known for controlling transcription by PARylating a panel of chromatin-related and transcription factors [13]. For example, the association of the oncogene SMAD3 with DNA is repressed by PARylation [14, 15]. However, the impact of PARylation on SMAD-driven gene transcription, tumor progression and metastasis [16, 17] remains unknown. Through deepening our understanding of the cellular processes dependent upon protein PARylation, and interrogating the possibility that protein PARylation is deregulated in cancers, more effective targeting of this pathway might be achieved. In particular, there is evidence that PARG plays an important role in promoting oncogenic effects, but this has not been widely explored.

Previous work showed that PARylation of the tumor suppressor CTCF is lost at late-stage breast cancer [18], indicating deregulation of the PARylation pathway. More recently, it was shown that the mono-(ADP-ribose) subunits produced during PARylation hydrolysis by PARG were subsequently metabolized to generate adenosine triphosphate (ATP) [19]. This energy fueled proliferation induced by the ER, supporting a potential oncogenic role for PARG. We now demonstrate that PARG acts as a classical oncogene, independent of ER status. Numerous in vitro studies showed that PARG depletion sensitized tumor cells to chemotherapy, radiation and DNA damaging agents, highlighting its therapeutic potential, but without translating this knowledge to relevant in vivo models.

In this study, we reveal that elevated PARG expression correlates with a poor prognosis for patients harboring invasive breast cancers. Consistent with this, we show that elevated PARG levels potentiate oncogenic transformation and invasion in vitro. In vivo, we find that PARG cooperates with the HER2 oncogene to promote tumor initiation and outgrowth. These oncogenic functions of PARG are seemingly dependent on its catalytic activity. Importantly, abrogation of PARG expression leads to decrease in tumor growth and metastasis of triple-negative models of breast cancer. Mechanistically, we find that PARG ablates SMAD2/3 PARylation, enhances the expression of SMAD target genes and that SMAD2/3 knockdown diminishes PARG-induced transformation and invasion. Overall, we demonstrate for the first time that elevated PARG activity is oncogenic and modulates SMAD activity, and we propose that PARG represents a potent target for anti-cancer therapy.

## Results

### PARG is overexpressed in breast tumors

**Fig. 1:**
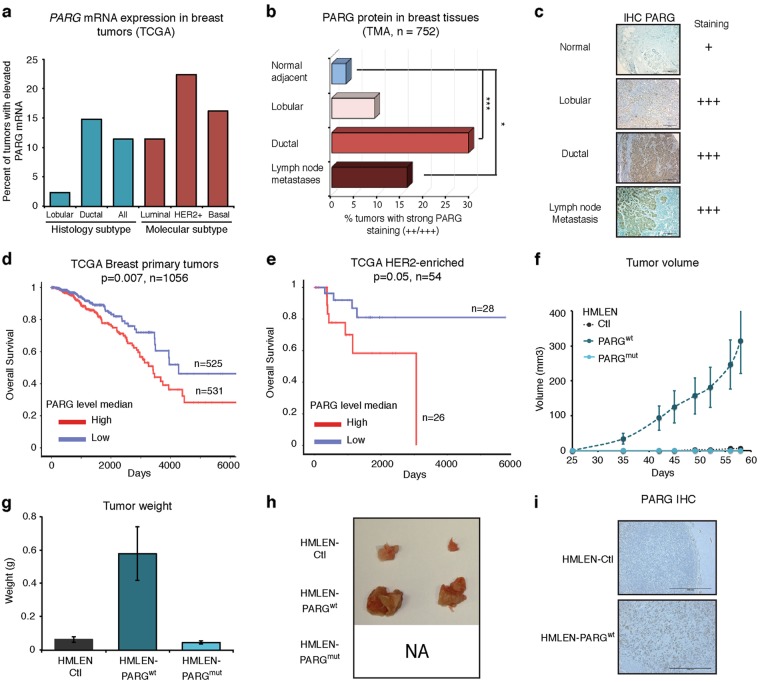
PARG expression is elevated in breast cancer and potentiates the tumor growth of HER2 expressing cells. **a** Analysis of TCGA breast invasive carcinoma database for PARG mRNA (*n* = 1215). Data were used to calculate the percent of tumors having elevated PARG mRNA levels. Breast cancer samples were classified according to molecular subtypes and assessed for PARG mRNA levels again using TCGA RNA-Seq data. **b** Bar graph recapitulating the percent of TMA cores with high PARG staining. *P* values were calculated by two-tailed *t*-test. ***P < 0.001, *P < 0.05. **c** Representative IHC staining for PARG. **d**, **e** Kaplan–Meier plots based on TCGA PARG mRNA. Scale bar 500 µm. **f** Growth curve of HMLEN tumors harboring vectors carrying Ctl, PARGwt or PARGmut. **g** Bar graph showing the weight of the tumors for each group (Ctl, PARGwt and PARGmut). **h** Representative photos of the tumors described in (**f**). **i** Representative immunohistochemistry staining for PARG in tumors from (**f**). Scale bar 500 μm

Database analysis of sequencing data from The Cancer Genome Atlas (TCGA) revealed that *PARG* is overexpressed across numerous tumor types (Figure S1a). For breast tumors, patients were further separated by histological criteria or molecular subtype. Approximately 15% of all invasive ductal breast tumors showed elevated *PARG* messenger RNA (mRNA) level, with the frequency being 20% in HER2-enriched and basal-like subtypes. Heightened *PARG* mRNA is five times more prevalent in invasive ductal carcinoma than invasive lobular carcinoma (Fig. 1a). To confirm these results at the protein level, we carried out immunohistochemistry (IHC) using a validated antibody against PARG (Figure S1b) on breast tissue microarrays (TMAs), including normal adjacent tissue. Overall, tissues from 752 subjects were stained (normal: *n* = 65, invasive ductal carcinoma: *n* = 530, lobular carcinoma: *n* = 108, lymph nodes metastases: *n* = 49). In normal tissue, little PARG protein was observed, with only 3% of the samples showing high intensity staining. On the other hand, 30% of the invasive ductal carcinoma tissues and 15% of lymph nodes metastases showed an intense PARG protein signal (Fig. 1b, c). Using a panel of 64 breast tumor cores, we tested for a correlation between Ki-67 staining and elevated PARG, but no association between high PARG and the proliferation marker was observed (data not shown). Overall, these results show that PARG expression is deregulated in breast tumor tissues, and it appears that high PARG protein is observed approximately fivefold more frequently in tumor tissues than normal epithelium.

### Elevated PARG is associated with poor overall survival

Next, to interrogate the possible clinical relevance of PARG in breast cancer, we evaluated the association of *PARG* mRNA levels with patient overall survival, first incorporating all breast cancer patients (Fig. 1d), followed by segregation into molecular subtypes. Simple separation of patients into high and low *PARG* expression, based on median *PARG* mRNA levels, revealed that *PARG* is significantly associated with poor overall survival in the total patient population. Based on molecular subtyping, high *PARG* levels correlated with poor overall survival in HER2-enriched and basal-like tumors (Fig. 1e and Figure S1c respectively), based on TCGA data, without showing any association in patients with luminal A/B subtypes (Figure S1d, e). We confirmed the same trend on the protein level (Figure S1f, g) in a cohort of HER2-enriched tumors versus ER+ tumors, but the number of HER2 + samples on this tumor microarray was too limited to test for statistical significance. The association of elevated PARG with a poor prognosis is consistent with potential oncogenic activity.

### PARG and HER2 act in synergy to promote tumor growth

To identify relevant cellular models to examine the functional significance of aberrantly high PARG expression, we screened a panel of human mammary cell lines for PARG protein expression. Consistent with our tissue staining, we observed that PARG protein levels were low in non-transformed cell lines including human mammary epithelial cells (HMLE) and MCF10A, but high molecular weight, nuclear PARG isoforms (~110 and 105 kDa) [20, 21] are often found highly expressed in transformed cell lines (Figure S1h). Genomic data and IHC staining indicated a potential oncogenic role for PARG in HER2-enriched tumors, and hence we decided to establish HMLE cell lines stably co-expressing both proteins. Here, we used ER-negative HMLEs [22] harboring stable expression of constitutively active HER2 (HMLEN) and infected them with empty vector (Ctl), full-length 110 kDa, wild-type PARG (PARG^wt^) or a catalytic inactive version of PARG E755/756A (PARG^mut^) (Figure S2a), as previously described [23]. HMLEN cells have a limited capacity to initiate tumors in vivo, and hence this represented an ideal model to examine the capacity of PARG to influence tumor initiation or outgrowth [24, 25]. We injected 4 × 10^6^ HMLEN-Ctl, HMLEN-PARG^wt^ and HMLEN-PARG^mut^ cells into the mammary fat pad of nonobese diabetic/severe combined immunodeficiency (NOD/SCID) mice. HMLEN initiate small tumors with incomplete penetrance, and the number and size of the tumors we obtained with HMLEN-Ctl cells were consistent with previously published data [25]. Of the mice harboring HMLEN-PARG^wt^ xenografts, 80% developed tumors of appreciable volume (~300 mm^3^), while only 40% of Ctl mice initiated palpable tumors (Fig. 1f–i). Interestingly, none of the HMLEN-PARG^mut^ xenografts developed palpable tumors, suggesting the mutant PARG may act as dominant negative in this context and repress HER2-driven outgrowth. Notably, HMLEN-PARG^wt^ tumors showed rapid outgrowth relative to HMLEN tumors, suggesting a synergy between HER2 and PARG, consistent with the correlation between high PARG mRNA levels and poor overall survival for HER2+ patients (TCGA data, Fig. 1e). Collectively, elevated expression of wild-type PARG in HMLEN increased tumor initiation rate and accelerated tumor growth in this ER-negative model, consistent with the role of PARG as an oncogene.

### PARG overexpression promotes cellular transformation in vitro and in vivo

**Fig. 2:**
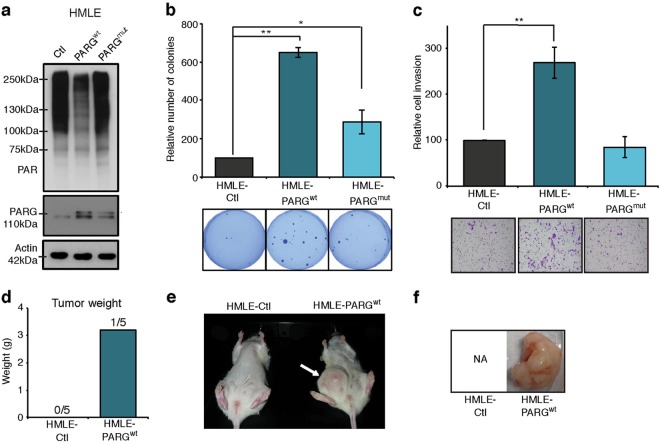
PARG overexpression in mammary epithelial cells promotes transformation and tumor growth. **a** Western blotting against PAR, PARG and actin carried out on protein extracts from HMLE infected with lentivirus carrying PARGwt, PARGmut or Ctl vector. **b** Soft agar colony formation assays using HMLE-Ctl, HMLE-PARGwt and HMLE-PARGmut cell lines. Representative pictures of each cell line are shown. Percent of colonies growing on soft agar relative to HMLE-Ctl. Error bars represent means ± s.e.m. of three independent experiments. *P* values were calculated by two-tailed *t*-test. **P < 0.01, *P < 0.05. **c** Invasion assays in Boyden chambers with matrigel using HMLE-Ctl, HMLE-PARGwt and HMLE-PARGmut. Percent of cells invading through matrigel relative to HMLE-Ctl. Error bars represent means ± s.e.m. of three independent experiments. *P* values were calculated by two-tailed *t*-test. **P < 0.01. **d** Bar graph showing the weight of HMLE-PARGwt tumor. **e** Picture of NOD/SCID mice injected with HMLE-PARGwt on the right inguinal mammary gland with HMLE-Ctl on the left. **f** Image of HMLE-PARGwt tumor

We subsequently investigated whether PARG alone could transform HMLE cells. Stable cell lines expressing control vector (Ctl), full-length, 110 kDa, wild-type PARG (PARG^wt^) and an E755/756A PARG mutant (PARG^mut^) were generated (Fig. 2a, Figure S2b). PARG^WT^ enzymatic activity was demonstrated through the reduction of protein PARylation under basal conditions (Fig. 2a). It might be noted that an array of PARylated protein species were observed across molecular weight ranges suggesting low intrinsic PARG activity. This is consistent with other reports showing a range of PARylated species in normal tissues such as mouse embryonic fibroblasts and nervous tissue [26, 27]. Specificity of the antibody was demonstrated through reduction of PARylation after exposure to PARP inhibition and extension of PAR chains after treatment with doxorubicin (Figure S2c, d). As expected, the expression of catalytically inactive PARG^mut^ protein did not catabolize protein PARylation to a detectable level, thus validating our constructs. While western analysis of the mutant PARG appeared to show reduced expression relative to wild-type PARG, probing against the FLAG tag revealed highly congruent expression levels (Figure S2b), suggesting the western antibody has a weaker affinity for the catalytically inactive mutant. PARG-expressing mammary epithelial cells gained the capacity for anchorage-independent growth and invasion through matrigel, without modulating the proliferation rate (Fig. 2b, c and Figure S2e). To ensure that the oncogenic effect observed in PARG-expressing cells was not due to disruption of another gene during cell infection, we depleted PARG in the HMLE-PARG^wt^ cells using short hairpin RNA (shRNA) and observed a reduction of the capacity of the cells to invade matrigel (Figure S2f-h). This phenomenon was also recapitulated in a p53/RB-null MCF10A cell line, where the introduction of PARG also led to increased invasiveness (Figure S2i-k). In agreement with our in vivo observations, the catalytic activity of PARG was needed to achieve significant levels of anchorage-independent growth (Fig. 2b). We also observed that ectopic PARG expression enhanced the anchorage-independent growth of HMLEN cells (Figure S2l, m). Next, we tested whether PARG alone would facilitate tumor initiation from HMLE cells. Following orthotopic injection of the Ctl and PARG^wt^ HMLE-derived cell lines into the mammary fat pad of NOD/SCID mice, we observed tumor growth only in the PARG^wt^ group (Fig. 2d–f), albeit with an extended latency of approximately 70 days (data not shown), consistent with the low penetrance and long latency observed in HMLE cells expressing other oncogenes including mutant H-Ras [28]. As expected, no tumors grew from the non-transformed HMLE-Ctl cells (Fig. 2d–f). Our data, described in Fig. 1f–i, using HMLEN cells expressing constitutively active HER2, showed a similar potency for PARG in promoting tumor outgrowth (HER2 40% vs PARG 20%). Together, these findings support the pro-oncogenic function of PARG that is largely dependent on its enzymatic activity.

### PARG depletion suppresses oncogenic phenotypes in vitro

**Fig. 3:**
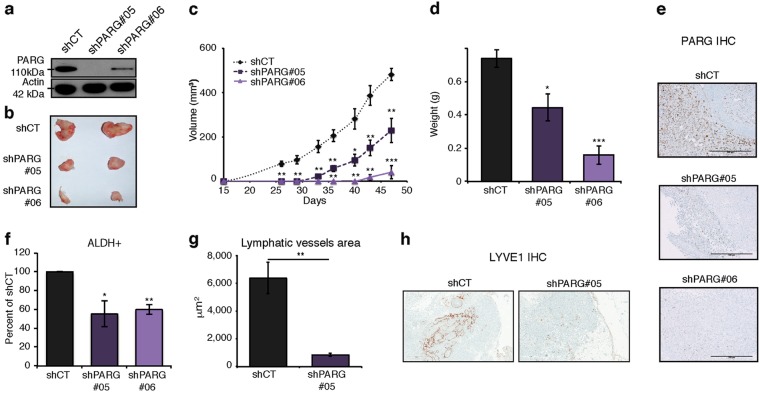
PARG depletion decreases tumor initiation and outgrowth. **a** Western blot showing PARG knockdown in MDA-MB-231-M2 cells. **b**, **c** Images of representative tumors from each group and volume of MDA-MB-231-M2 tumors in NOD/SCID mice+/– PARG knockdown. Error bars represent mean ± s.e.m. *P* values were calculated by two-tailed *t*-test. **P < 0.01, ***P < 0.001. **d** Tumor weight at endpoint of experiment shown in (**b**). *P* values were calculated by two-tailed *t*-test. *P < 0.05, ***P < 0.001. **e** Representative PARG IHC on tumors from (**b**). Scale bar 500 μm. **f** Bar graph representing the proportion of ALDH+ cells in MDA-MB-231-M2 and PARG knockdown populations as measured by flow cytometry. Error bars represent mean ± s.e.m. *P* values were calculated by two-tailed *t*-test. *P < 0.05, **P < 0.01. **g** Average lymphatic vessel area measured based on LYVE1 staining. Error bars represent mean ± s.e.m. *P* values were calculated by two-tailed t-test. **P < 0.01. **h** Representative LYVE1 IHC images. Scale bar 500 μm

The data described above suggest that inhibition of PARG enzymatic activity could have therapeutic implications. To test this hypothesis, we employed lentiviral constructs expressing shRNA targeting *PARG* (shPARG#05 and shPARG#06) (Fig. 3a). We first utilized MDA-MB-231 cells because they show high 110 kDa PARG isoform expression (Figure S1g) and represent a well-characterized model of basal-like breast cancer; a subtype of breast cancer where high PARG expression correlates with poor overall survival (Figure S1b). PARG depletion prohibited aggressive oncogenic phenotypes including reduction of cell migration, loss of invasiveness and inability to grow in an anchor-independent manner (Figure S3a-g).

### PARG depletion impairs tumor initiation and growth in vivo

We next tested whether *PARG* depletion would affect tumorigenesis in vivo. Control shRNA (shCT) or sh*PARG* transduced MDA-MB-231 cells were injected into mammary fat pad of NOD/SCID mice. In the first experiment, 0.5 × 10^6^ cells were injected and here, only mice receiving shCT cells showed measurable tumor growth (5/5 shCT, 0/5 shPARG#05; Figure S3h). After increasing the number of cells injected to 2 × 10^6^, we were able to obtain palpable tumors growth in all groups, possibly through enriching the number of tumor-initiating cells (TICs) in the population (shCT: 100%, shPARG#05: 80%, shPARG#06: 40%, Fig. 3a–c and data not shown). While the PARG knockdown tumors often grew to palpation in these experiments, we consistently observed that MDA-MB-231 cells depleted of *PARG* were significantly delayed in tumor outgrowth (Fig. 3b–e). TICs have been proposed to be responsible, at least partially, for promoting neoplastic growth and metastasis [29]. Based on our in vivo data (Figs. 1, 2 and Figure S3h), PARG levels appear to enhance the capacity of transformed cells to initiate tumors, suggesting a role for PARG in regulating the number of TICs. One of the hallmarks of TICs is an increase in aldehyde dehydrogenase (ALDH) activity [29, 30]. We observed a 50% decrease in the number of MDA-MB-231 cells positive for ALDH activity when PARG was depleted (Fig. 3f), possibly explaining the lack of tumor initiation observed in vivo for PARG-depleted cells. One theory for the origin of metastatic lesions, based on multiple models, is that TICs disseminate from the primary tumor and generate neoplastic growth at distal sites [30, 31]. A critical step during metastasis is the intravasation of malignant cells from the primary tumor into the blood or lymphatic vessels. We performed IHC using the lymphatic vessel marker LYVE1 on tumors derived from MDA-MB-231 cells expressing shCT or sh*PARG*. PARG-depleted tumors showed a dramatic decrease in the density and area of lymphatic micro-vessels (Fig. 3g, h). These findings led us to further characterize the role of PARG in potentiating metastasis.

### PARG depletion abrogates metastasis

**Fig. 4:**
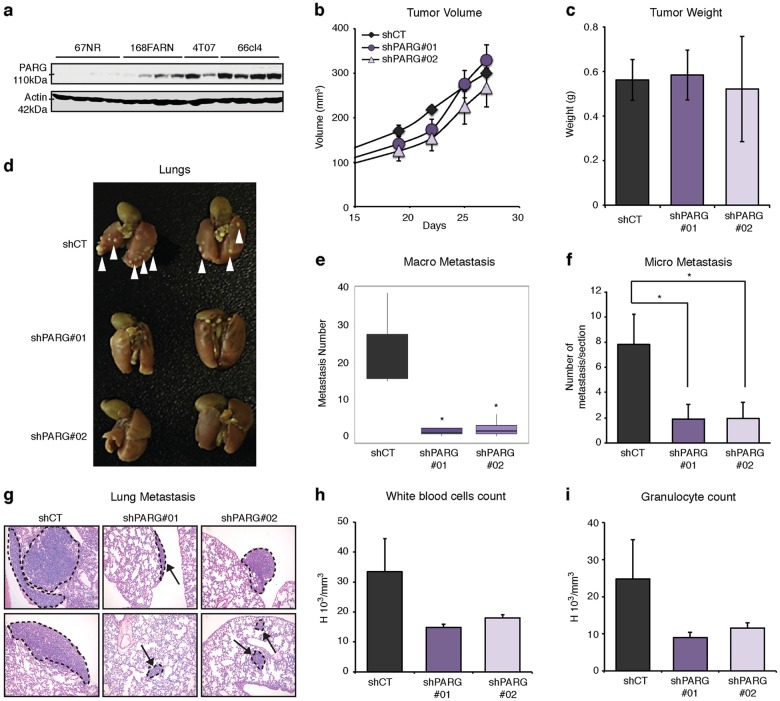
PARG depletion impairs the metastatic potential of 66cl4 tumors in an orthotopic syngeneic transplantation model. **a** Western blotting against PARG and Actin were carried out on proteins extracts from 67NR and derivative tumors. **b** Tumor volumes of 66cl4 orthotopic xenografts+/– PARG knockdown. Error bars represent mean ± s.e.m. **c** Tumor weights at the endpoint of the experiment shown in (**b**); error bars represent mean ± s.e.m. **d** Images of lungs from mice with 66cl4 control, or PARG knockdown tumors, showing macrometastases after Bouin staining. **e** Quantification of metastatic surface nodules from experiment shown in (**d**). *P* values were calculated by two-tailed *t*-test. *P < 0.05. **f** Quantification of micro-metastases in lung cross sections from 66cl4 xenograft mice metastasis. Average number of metastasis per 4 μm section. Error bars represent mean ± s.e.m. *P* values were calculated by two-tailed *t*-test. *P < 0.05. **g** H&E staining of lung 4 μm cross-sections quantified in (**f**) showing representative metastasis images for each group. **h**, **i** Total white blood cell counts and granulocyte counts quantified at the endpoint of experiment shown in (**b**, **c**)

To explore a role for PARG in metastasis, we took advantage of a well-characterized panel of isogenic murine breast cancer cell lines that differ in their ability to metastasize when implanted into the mammary fat pads of syngeneic mice [32]. These include 67NR (non-metastatic), 168FARN (metastatic to lymph nodes), 4TO7 (weakly metastatic to the lungs) and 66cl4 (highly metastatic to the lungs). We observed that PARG was absent from 67NR-derived tumors, but its expression increased in the more aggressive tumors (Fig. 4a). First, the impact of PARG depletion in 66cl4 cells was investigated in vitro. PARG repression led to a decrease in migration and invasion (Figure S4a-d) similar to what we had previously observed in MDA-MD-231 cells. Because the non-metastatic cell line 67NR expresses PARG at nominal levels, we reasoned that PARG was not driving tumor growth in this model. Thus, we hypothesized that the increased PARG expression seen in the more aggressive 67NR-derivative cells, such as 66cl4, may serve to promote tumor dissemination and metastasis, but likely not tumor outgrowth. 66cl4 cells were infected with control shRNA, or those targeting *PARG*, then injected orthotopically into syngeneic BALB/c mice. As expected, little difference in tumor initiation and outgrowth was observed between the shCT and sh*PARG* groups (Fig. 4b, c). The number of macroscopic surface metastases on the lungs of the *PARG*-depleted group was strikingly decreased with a median value of 3 metastases per lung compared to 20 tumor nodules on the lungs in the control group (Fig. 4d, e). Serial step sectioning of the lungs followed by hematoxylin and eosin (H&E) staining to more robustly quantify micro-metastasis revealed that both the number and size of metastases (Fig. 4f, g) were considerably reduced in the PARG knockdown cohort. Using this model, it was previously found that increased white blood cell counts, especially granulocytes, are associated with the presence of metastasis [33]. As expected, we observed 2–3-fold higher numbers of total white blood cells and granulocytes in mice injected with control 66cl4 cells versus those with reduced PARG (Fig. 4h, i). Overall, these results highlight a key role for PARG in driving metastasis.

### PARG modulates epithelial-to-mesenchymal transition

**Fig. 5:**
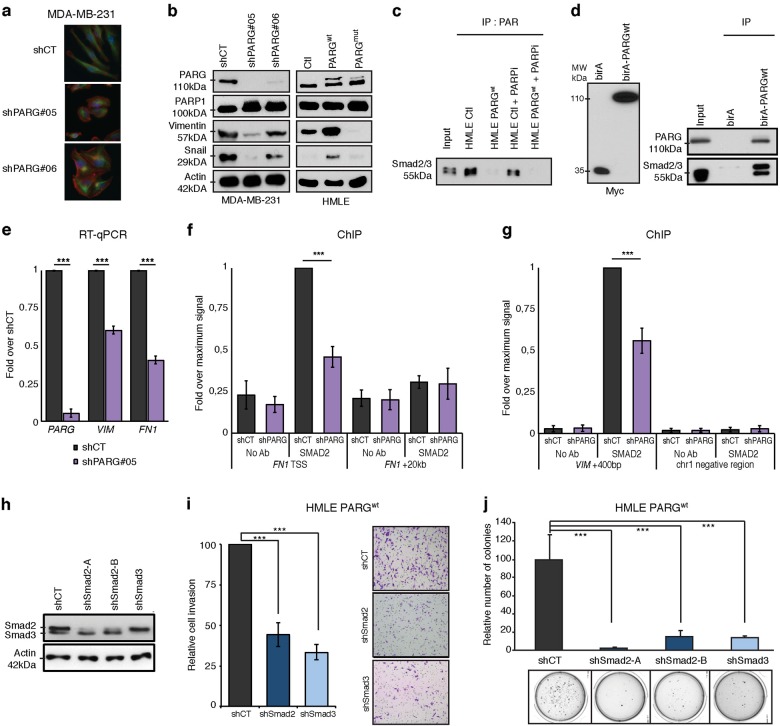
Depletion of SMAD2 or SMAD3 abrogates PARG-induced transformation of HMLEs. **a** IF of Vimentin (green) and phalloidin (red) in MDA-MD-231 control and PARG knockdown cells. **b** Western blot of PARG, Vimentin, Snail and Actin in HMLE-Ctl, HMLE-PARGwt, MDA-MB-231 shCT and MDA-MB-231 shPARG#05. **c** Co-IP of SMAD2/3 using anti-PAR antibody in HMLE-Ctl, HMLE-PARGwt, HMLE-Ctl+PARPi and HMLE-PARGwt+PARPi. **d** HEK293T were transfected with birA, birA-PARGwt or birA-PARGmut and follow by WB of PARG and SMAD2/3. **e** RT-qPCR for VIM, FN1 and PARG using mRNA from cells listed in (**b**). Error bars represent mean ± s.e.m. of three independent experiments. *P* values were calculated by two-tailed *t*-test. ***P < 0.001. **f** ChIP for SMAD2 association with the FN1 gene in MDA-MB-231-M2 cells+/– PARG knockdown. TSS represents “transcription start site”. FN+ 2 kb represents a region negative for SMAD2 binding. *P* values were calculated by two-tailed *t*-test from three independent experiments. ***P < 0.001. **g** ChIP showing the association of SMAD2 at the VIM proximal promoter region in MDA-MB-231-M2 cells+/**-** PARG knockdown. *P* values were calculated by two-tailed *t*-test from three independent experiments. ***P < 0.001. **h** WB against SMAD2/3 and actin on HMLE-PARGwt following knockdown of SMAD2 and SMAD3. **i** Invasion assay using HMLE-PARGwt cells following SMAD2 or SMAD3 knockdown. Percent of cells invading through matrigel relative to HMLE-PARGwt-shCT. Error bars represent mean s.e.m. of three independent experiments. *P* values were calculated by two-tailed *t*-test. ***P < 0.001. **j** Soft agar assays using HMLE-PARGwt cells following SMAD2 or SMAD3 knockdown. Percent of colonies growing on soft agar relative to HMLE-PARGwt-shCT. Error bars represent mean s.e.m. of three independent experiments. *P* values were calculated by two-tailed *t*-test. ***P < 0.001

During our characterization of *PARG*-depleted MDA-MB-231 cells, we noticed that *PARG* knockdown, using two independent shRNAs, resulted in MD-MB-231 cells losing their characteristic spindle/mesenchymal shape, and acquiring a more epithelial-like phenotype (Fig. 5a). The epithelial-to-mesenchymal-like transition (EMT) has been shown to drive carcinogenesis and metastasis and is potentially associated with TICs [24, 34, 35]. Thus, we decided to investigate a role for PARG in an EMT because this represents a plausible mechanism explaining the phenotypic changes we observed in vitro and in vivo following modulation of PARG. First, we looked at the impact of modulating PARG protein level on the expression of the mesenchymal protein Vimentin and the EMT-inducing transcriptional regulator Snail in HMLE and MDA-MB-231 cells (Fig. 5a, b). PARG-depleted MDA-MB-231 cells expressed less Vimentin and Snail, consistent with a gain in an epithelial phenotype. Conversely, ectopic PARG expression in human mammary epithelial cells (HMLE) increased Vimentin and Snail levels (Fig. 5b). Likewise, elevated PARG promoted Snail expression in P53/RB-null MCF10A cells (Figure S2g-i). These changes in gene expression were not recapitulated using a catalytically inactive PARG mutant (Fig. 5b), indicating changes to protein PARylation was driving this EMT-like transition. EMT may be stimulated via signal transduction through the transforming growth factor-β (TGFβ) pathway. A critical downstream event in this pathway is the formation of heterodimeric SMAD2/3–SMAD4 complexes, which drives the transcription of EMT inducers such as *VIM* and *SNAI1* [16, 17]. Therefore, we next examined the potential of PARG to influence the targeting of SMAD2/3 to target genes.

### PARG regulates SMAD2/3 DNA binding activity

Previous work showed that SMAD3/4 PARylation impedes heterodimeric SMAD complexes from binding to chromatin [14, 15]. SMAD3 is PARylated at glutamic acids 50 and 52 within a “DELEK” motif, which is conserved to lower vertebrates. The “DELEK” motif is found at position 59–63 of SMAD2, and thus SMAD2 is also very likely to be PARylated. We hypothesized that PARG interacts with PARylated SMAD complexes within the nuclei to impair its capacity to bind DNA. Indeed, using an anti-PAR antibody (clone 10H) to IP PARylated proteins, we found that SMAD2/3 is PARylated in HMLE-Ctl cells, but this post-translational modification of SMAD2/3 was largely lost in HMLE-PARG^wt^ cells (Fig. 5c). Exposure to the PARP inhibitor Olaparib for 24 h reduced SMAD2/3 PARylation, but surprisingly, unlike PARG overexpression, Olaparib did not completely ablate SMAD2/3 PARylation. This may be due to compensation by other PARPs such as PARP5a/b, or may reflect a long half-life of SMAD PARylation in this cell type. To further examine an interaction between PARG and SMAD2/3, we transfected cells with birA-tagged PARG and subsequently probed SMAD2/3 for biotinylation. Strepavidin beads captured little SMAD2/3 in cells transfected with BirA alone (Fig. 5d). In contrast, we observed that both SMAD2 and SMAD3 were robustly pulled down from PARG^wt^-birA transfected cells (Fig. 5d), indicating that SMAD2/3 interacts with PARG. As opposed to PARylation, phosphorylation of SMAD2/3 remained constant in response to changes in PARG levels (Figure S5a, b), indicating that modulation of signal transduction events downstream of TGFβ receptors is unlikely to represent a mechanism whereby PARG controls the activity of SMADs. PARG knockdown results in a significant decrease of *FN1* and *VIM* mRNA in MDA-MB-231 cells (Fig. 5e). So we next evaluated the impact of PARG depletion on the association of SMAD2/3 with chromatin at these target genes by chromatin immunoprecipitation (ChIP). We see that PARG depletion reduced SMAD2 binding by 50% at *FN1* and *VIM* promoters (Fig. 5f, g). In contrast, in HMLE, we observed that ectopic expression of PARG augmented the TGFβ induction of vimentin and enhanced SMAD3 recruitment to the *VIM* immediate upstream region following TGFβ treatment (Figure S5c, d). In contrast, no effect on SMAD3 binding was observed when the E755/756A PARG mutant was expressed (Figure S5e). Collectively, we show that PARG interacts with SMAD2/3 leading to their hypoPARylation, and thereby promoting transcriptional activity of key EMT regulators.

### PARG-induced transformation is dependent on SMAD2/3

We next interrogated whether SMAD activity is required for the pro-oncogenic effects mediated by PARG. If SMAD2/3 are downstream effectors of PARG, then we expect SMAD2/3 knockdown to abrogate the phenotypic change observed in HMLE-PARG^wt^ cells. Depletion of SMAD2 or SMAD3 in HMLE-Ctl or HMLE-PARG^wt^ (Figure S5f) did not significantly alter cell proliferation but dramatically reduced the capacity of the HMLE-PARG^wt^ cells to invade through matrigel or to grow in an anchorage-independent manner (Fig. 5h–j, Figure S5g, h). These results confirmed that the pro-oncogenic effects of PARG are mediated, in part, through SMAD2/3 activity.

## Discussion

Modulating the PARylation pathway as an approach to anti-cancer therapy has been an area of intensive investigation in recent years, especially inhibition of PARylation synthesis through the use of PARP1/2 inhibitors. However, regulation of protein PARylation in cancer remains understudied, as does the potential of targeting members of this pathway beyond PARP1/2. Classical studies demonstrate that Parp1^–/–^ mice develop spontaneous tumor formation in various tissues including the mammary gland [36] and are prone to carcinogen-induced neoplastic growth [37]. Further, the loss of PARP1 exacerbates lymphogenesis in DNA-PK-null mice [38] and promotes hepatocellular carcinoma in Ku80 heterozygous mice [39]. It remains unknown whether SMAD2/3 binding profiles are altered in PARP1^–/–^ mice, but future studies will be carried out to uncover whether altered SMAD activity contributes to the tumorigenic phenotypes observed in PARP1^–/–^ mice. It will also be relevant for future studies to utilize PARP1^–/–^ mice to map the genomic binding profiles of additional transcription factors, such as the oncogene FOXC1, known to be modified by PARylation [40]. Data from these PARP1-deficient mouse models provide strong evidence that diminishing protein PARylation is oncogenic in nature and indicate that increased activity of enzymes abrogating protein PARylation, such as PARG, might confer an oncogenic phenotype. Here, we show that PARG expression is elevated in breast cancer and that aberrantly high PARG levels support transformation, tumor outgrowth and metastasis.

PARG hydrolyzes PAR chains into mono (ADP-ribose) subunits that can be subsequently metabolized to generate ATP [19]. A recent publication demonstrated that increased PARG activity supplies ATP for nuclear hormone receptor-mediated chromatin remodeling, leading to the expression of ER target genes and cellular proliferation [19].

Here, we find that elevated PARG activity in ER-negative HMLE cells does not impact proliferation, yet promotes neoplastic growth, suggesting an ER-independent mechanism of transformation. This is consistent with patient data correlating PARG expression with poor prognosis in ER-negative triple-negative and HER2+ breast cancers.

To date, most studies probing the cellular functions of PARG have focused on its key role in DNA damage repair pathways [21, 41–43]. However, it has long been known that PARG regulates transcription through multiple mechanisms [14, 44]. Here, we highlight that PARG enhances the transcription of EMT-related genes via potentiating the association of hypoPARylated SMAD2/3 with chromatin. This function of PARG appears to play a central role in mediating anchorage-independent growth and invasion, because the absence of SMAD2 or SMAD3 repressed these oncogenic phenotypes. However, we propose that PARG also stimulates neoplastic growth via mechanisms beyond SMAD2/3 activation. Transgenic mice expressing an activated HER2/NEU showed an enhanced frequency of lung metastasis when crossed with mice expressing activated TGFβ type I receptor within the mammary epithelial compartment [45, 46]. In this model, neither tumor initiation nor outgrowth driven by the NEU oncogene was exacerbated by TGFβ signaling. In our HMLE model, PARG clearly enhances HER2-driven tumorigenesis, suggesting additional modes of crosstalk with the HER2 pathway. Moving forward, we hypothesize that PARG modulates cell metabolism to provide HER2 overexpressing cell with a growth advantage.

PARG-mediated catabolism of PARylation chains leads to a subsequent accumulation of mono-ADP-ribose (mADPr) [47]. mADPr is utilized for NAD+ synthesis through the “NAD+salvage pathway”, largely dependent on the enzyme NAMPT [48]. NAD+ is an essential cofactor for energy production through the tricarboxylic acid (TCA) cycle. It is well known that the reduction of NAD+ to NADH within the mitochondria provides electrons for the electron transport chain, which in turn, powers ATP synthesis. NAD+ may also modulate glucose metabolism through regulating Sirtuin activity. In turn, glucose catabolism feeds the TCA cycle with pyruvate [49]. Interestingly, it has been shown that PARylation may directly impair glycolysis by inhibiting the catalytic activity of Hexokinase [49]. It therefore is reasonable to expect that elevated PARG activity may increase the total store of energy available to the cell for growth and survival, and may further enhance glycolysis. The potential for PARG to enhance energy production may be particularly important within tumors where nutrients are often limited. Based on these studies, and other reports showing glycolytic flux is required for HER2-driven tumor growth [50], we propose that potentiating energy production represents another mechanism whereby PARG acts as an oncogene.

Overall, based on the pro-tumorigenic effects of PARG activity, and the profound effect of PARG knockdown on tumor growth and metastasis, our data emphasize the potential of PARG inhibition as a rational approach to drugging the PAR pathway, beyond PARP1/2 inhibition, for anti-cancer therapy.

## Materials and methods

### Cell lines and cell culture

The cell lines (MCF7, HEK293T, T47D) were maintained in Dulbecco's modified Eagle's medium (DMEM; Wisent) containing 10% fetal bovine serum (FBS, VWR). MDA-MB-231, MDA-MB-231-M2 and 66cl4 were grown in RPMI medium (Wisent)+10% FBS. MDA-MB-231-M2, a highly metastatic variant of MDA-MB-231 cells, was isolated from lung nodules induced by the parental MDA-MB-231 [51]. The HMLE cell line was a generous gift of Dr. R Weinberg’s laboratory (Whitehead Institute) and maintained in EpiMax medium (Wisent)+supplements and 10% FBS [28]. MCF10A TP53^–/–^ RB1^–/–^ was grown in DMEM/F12 (50:50), 2% horse serum, epidermal growth factor 20 ng/ml, hydrocortisone 0.5 mg/ml, insulin 10 μg/ml and cholera toxin 100 ng/ml. All cell lines were tested for mycoplasma contamination.

### Chemicals, reagents and virus production

For shRNA directed knockdown, sequences targeting PARG, SMAD2 or SMAD3 were carried in pLKO.1-puro lentiviral vectors (Sigma). Their targeting sequences were listed in Table S1. PARG-expressing constructs were bought from GeneCopoeia in Lentiviral vectors Lv102, Lv216 and Lv166. For the bioID experiment, PARGwt and PARGmut (E755/756A) were cloned in the pcDNA3.1-myc-BirA (R118G) vector. All the antibodies used in this publication are listed in Table S2.

Lentiviral particle packaging and transduction was carried out as we have recently described [52]. The cells were transduced with lentivirus in the presence of polybrene (8 μg/ml) for 48 h. For knockdown using pLKO vector, new infections were carried out for each experiment.

### Reverse transcription and quantitative real-time PCR (RT-qPCR)

Total RNA was extracted from cultured cells using GenElute (Sigma) and reverse transcribed using an all-in-one kit (ABM) with random hexamer. Quantitative PCR (qPCR) was performed using SYBR Green PCR master mix reagent (Promega) with a 7500 Fast real-time system (Applied Biosystem). The threshold cycle (Ct) value for each gene was normalized to the Ct value for 36B4. The RT-qPCR primer sequences are listed in Table S3.

### Murine models

For xenograft studies using human HMLE or MDA-MB-231-M2 cell lines, 5-week-old female NOD/SCID mice were utilized from The Jackson laboratory. For the syngeneic experiments with 66cl4 cells, 5-week-old female BALB/c mice were employed (Jackson). Cell suspensions in phosphate-buffered saline (PBS) were injected into the left inguinal mammary fat pad of the mice. Tumor growth was monitored twice a week for the length of the experiment using digital calipers. Tumor volume was calculated using the formula *V* = 1/2 × length (mm) × width (mm)^2^ as we previously described [53]. At time of killing, primary tumor and lungs were recovered and primary tumors were washed with 1× PBS followed by flash freezing for half the tumor and the other half was fixed in 10% formalin overnight and then embedded. Prior to embedding, lungs were fixed in Bouin’s solution overnight and then visible metastases were counted. The number of micro-metastases per lung was determined using 5 step-wise sections of 50 μm and stained with H&E. All animal experiments and number of animals chosen were in accordance with the Canadian council on animal care guidelines. No statistical method was used to determine the sample size. All animals injected with cells were included in the analysis. No randomization or blinding was used.

### Plasma analyses

Cardiac puncture was performed on mice at tumor endpoints. Blood was collected in EDTA-containing tubes and total blood cell counts were performed using a Scil Vet abc Hematology Analyzer (Scil Vet Novations).

### Western blot

Cells were harvested at 70–85% confluency and washed with PBS. Next, 2 volume of whole cell lysis buffer (20 mM Tris pH 7.5, 420 mM NaCl, 2 mM MgCl_2_, 1 mM EDTA, 10% glycerol, 0.5% NP-40, 0.5% Triton, 1 mM DTT, protease inhibitor, 2 mM PMSF, 2 mM NaF and 10 mM BGP) was added to the pellet and then placed on ice for 30 min. The lysate was cleared by centrifugation at 13,000 rpm and protein concentration was quantified by Bradford protein assay. Per gel 20 μg of protein was loaded.

### Cell migration and invasion assay

Cell migration and invasion was measured in a Boyden chamber system according to standard protocols [54]. MDA-MB-231, 66cl4 or HMLE cells were starved in their respective media containing 1% FBS for 24 h. For migration assays, HMLE cells (7.5 × 10^5^) were placed in the upper chamber with non-coated membrane (12-well insert; 8-um pore size; Corning Inc.). For invasion assay, MDA-MD-231 and 66cl4 cells (5 × 10^5^) and HMLE cells (7.5 × 10^5^) were placed in the top chamber with matrigel-coated membrane (300 μl of matrigel was added at 300 μg/ml for the MDA-MB-231 and 66cl4, and at 30 μg/ml for the HMLE; 12-well insert; 8 μm pore size; Corning Inc). In both assays, cells were plated in 1 ml of serum-free medium in the top chamber, and the lower chamber was filled with 1.5 ml of complete media. The total number of cells that migrated into the lower chamber was counted after 18 h of incubation at 37 C and 5% CO_2_. Cells that had not penetrated the filter were wiped out with cotton swabs, and cells that had migrated or invaded to the lower surface of the filter were fixed with 5% glutaraldehyde and then stained with 0.05% crystal violet. Fives pictures of each chambers were taken and cells counted. Three chambers by condition by experiment, and each experiment have been done three times independently.

### ALDH activity assay

ALDH activity was detected using ALDEFLUOR® staining kit (StemCell Technologies). Live cells were harvested after treatment and washed 2× with PBS. The cells were incubated in 100 μl of assay buffer+1 μl of 300nMaldefluor reagent and 1 ml of DEAB reagent for 30 min. A matching sample with DEAB added serves as background control. The 7-aminoactinomycin D staining is used to exclude necrotic cells during FACS analysis. The aldefluor intensity is measured using BD FACScalibur.

### Wound healing assay

MDA-MB-231 or 66cl4 cells were plated in 6-well plates and grown until they reached full confluence. A P200 tip was using to scratch through the cells in the well following a ruler as a guide. A photo was taken at *T* = 0 and another after 22 h. Then, using Infinity Analysis software (v5.0.2) the distance between the scratch borders was measured at 5 locations along the scratch. The average diameter of the scratch was calculated to discern differences in cell migration.

### Immunohistochemistry

IHC was carried out essentially as we previously described [55].

Immunohistochemical staining was performed at the Segal Cancer Center Research Pathology Facility. The anti-PARG antibody (NBP1-89450, Novus Biologicals) was validated using paraffin-embedded cell lines showing high and low PARG expression. Slides carrying TMA sections were then loaded onto the Discovery XT Autostainer (Ventana Medical System). All solutions used for automated immunohistochemistry were from Ventana Medical System unless otherwise specified. Slides underwent de-paraffinization with the EZ PREP solution (Ref# 950-100), heat-induced epitope retrieval with Cell Conditioning solution CC1 pH 8.0 (Ref# 950-224) at standard condition (60 min at 95 C). Briefly, rabbit polyclonal anti-PARG diluted at 1:25 in the antibody diluent (Ref# 251-018) was manually applied for 32 min at 37 °C and then followed by the detection kit. Slides were counterstained with hematoxylin for 4 min; blued with Bluing Reagent for 4 min, removed from the autostainer, washed in warm soapy water (Dawn) dehydrated through graded alcohols, cleared in xylene and mounted with Permount.

#### TMA description

Nine tissue microarrays (BR729, BR1503c, BR1504a, BR1921, BR2410, BR2411, BR10010b and BRC961, US Biomax, and one custom breast TMA carrying a total of 530 invasive ductal carcinoma, 108 lobular, 49 lymph nodes and 65 normal adjacent tissue) were probed for PARG protein expression. For the custom TMA, the use of human tissues for this study was approved by the Health Research Ethics Board of Alberta Cancer Committee (HREBA-CC) and carried out in accordance with the approved guidelines [56]. Tissue cores were from a cohort of 167 patients diagnosed with progressive breast cancer. After obtaining written informed consent from each patient, a TMA was constructed using a manual tissue arrayer (Beecher Instruments, Silver Spring, MD).

#### TMA staining quantification

Slides were analyzed blindly to the tissue type and clinical pathology and conducted by two independent certified pathologists. Each core was scanned in a low power field to choose the most stained area predominant in at least 10% of tumor cells. Protein expression was assessed using a four-tiered system (0, negative; 1, weak; 2, moderate; and 3, high expression). Each core was evaluated separately and a final score for each case was achieved by averaging the total intensity value of all cores within a specific patient sample. For survival and frequency analysis, samples were categorized into two groups: (N) negative/weak and (P) moderate/strong positive cases. For the tumor samples, only tumor tissue was taken into account for the score calculation.

### TCGA expression and survival analysis

TCGA breast invasive carcinoma RNA-seq expression data (IlluminaHiSeq) were downloaded from the cancer browser website (https://genome-cancer.ucsc.edu/) and the expression data were normalized to *z*-score [57]. Average PARG expression in normal tissue samples was used to calculate the percent of tumor samples with at least 1.5-fold higher expression beyond normal. For overall survival data were downloaded from the TCGA website. Tumor samples were split by the median and Kaplan–Meier survival curves were generated using SPSS 22.0.0 software. The *P* value associated with the Kaplan-Meier plot represents log-rank test (SPSS 22.0.0).

### bioID affinity capture

HEK293T cells at 30% confluency were transfected with birA constructs (5 μg for the birA only and 7 μg of birA-PARGwt and birA-PARGmut). On the following day, the media were changed and 50 μM of biotin was added for 18 h after which cells were washed with PBS and then 1 ml of whole cell lysis buffer was added. The lysate was sonicated for 2 × 15 s at 20% power (Sonic Dismembrator Model 500) and kept on ice for 30 min followed by centrifugation at maximum speed for 30 min. Then, 1.5–2 mg of proteins were immunoprecipitated with 20 μl of a 50% slurry of strepavidin Dynabeads rotating for 3 h at 4 C. After caper of biotinylated protein, beads were washed 3 times with whole cell lysis buffer, 2 times with TE and 3 times with 50 mM ammonium bicarbonate pH 8. Captures proteins were finally eluted with 50 μl of 2× Laemmli buffer and 10 min at 95 °C [58].

### Coimmunoprecipitation (co-IP)

To obtain protein for Co-IP, cells were treated or not with 1 μM of Olaparib for 24 h, then collected at 1000 rpm and washed once with 1× PBS, followed by addition of two volumes of whole cell lysis buffer as described above. Cells then sat on ice with occasional tapping for 30–45 min, spun at top speed in a microcentrifuge for 25 min after which the supernatant was transferred to a new tube. Then, 1–2 mg of protein was used for each IP. Protein lysates were diluted at least 5 times with IP Buffer (20 mM Tris pH 7.5, 50 mM NaCl, 10 mM MgCl_2_, 2 mM EDTA, 0.5% Triton and protease inhibitors). Preclearing was done with 50 μl of slurry of protein G beads 50% for 2 h. The beads were removed by centrifugation at 5000 rpm for 3 min and the supernatant was transferred to a new tube. Next, 2 μg of PAR antibody was added to capture PARylated proteins and nutated overnight at 4 °C. The next morning, 25 μl of 50% protein G beads were added and nutated for an additional 2 h. Beads were captured, washed 3 times with IP buffer and once with IP Buffer having Triton adjusted to 0.1%. Proteins were eluted by adding 25 μl of 2× Laemmli buffer and heated to 100 °C for 10 min. Tubes were then spun at high speed for 1 min to sediment beads, and the supernatant loaded onto polyacrylamide gels for electrophoresis.

### Immunofluorescence (IF)

IF was carried out as previously described [52]. Cells were seeded in 6-well plates containing four 12 mm circular coverslips per well. The cells were grown at least 2 days prior to fixation to ensure adequate adhesion of the cells on the coverslips. Cells were washed with 1× PBS and then fixed with 2% paraformaldehyde in PBS for 15 min at room temperature followed by two washes with 1× PBS. Cells are then permeabilized using 3% BSA/PBS/0.2% Triton for 10 min at room temperature followed by washing twice with 3% BSA/PBS after which the primary antibody was added overnight at 4 °C. After three washes, the secondary antibody was added for 1 h at room temperature (RT). Next, the coverslips were washed four times with the third wash carring DAPI (4′,6-diamidino-2-phenylindole) at 10 µM. Finally, the coverslips are mounted with prolong gold.

### Chromatin immunoprecipitation

ChIP assays and analyses were carried out similar to what we previously described [59] with some modification. Cells were collected and cross-linked with 1% formaldehyde in PBS for 10 min at RT. The cross-linking reaction was stopped with 125 mM glycine, and the cells washed with 1× PBS and stored at −80 °C until further processing. Cells pellets were then lysed and the DNA sheered by sonication in cell lysis/ChIP buffer (0.25% Nonidet P-40, 0.25% Triton X-100, 0.25% sodium deoxycholate, 0.1% SDS, 50 mM Tris (pH 8.0), 50 mM NaCl, 5 mM EDTA) 15 times for 15 s each (Sonic Dismembrator Model 500). Lysates were centrifuged for 15 min at 20,000 × *g* at 4 °C, and supernatant was collected. A total of 0.5 mg of protein was precleared for 2 h with Protein G agarose beads (50% slurry blocked with salmon sperm DNA) at 4 °C. IP was carried out by adding 4 µg of antibody and 30 µl of agarose G beads, nutating overnight at 4 °C. After IP, beads were pelleted by centrifugation, followed by four washes to remove unspecific binding using a variety of buffers with varying concentrations of salt. Buffers 1–3 contained 0.1% SDS, 1% Triton X, 2 mM EDTA, 20 mM Tris (pH 8.0), 150 mM NaCl, 300 mM NaCl, 500 mM NaCl, respectively. Buffer 4 contained 0.25 M LiCl, 1% Nonidet P-40, 1% sodium deoxycholate, 1 mM EDTA and 10 mM Tris (pH 8.0). Two additional washes with TE were done to remove any residual buffers from the beads. Complexes bound to the beads were eluted with 200 µl of elution buffer (1% SDS, 1 mM EDTA, 50 mM Tris (pH 8.0)) at 65 °C for 25 min. Beads were pelleted by centrifugation and supernatant was collected. Reverse cross-linking was done by adding 0.2 mM NaCl at 65 °C overnight followed by treatment with Proteinase K at 45 °C for 1 h, and a second incubation of 15 min at 65 °C. DNA recovery was carried out using QIAquick PCR purification kit according to the manufacture’s protocol. DNA was eluted in 100 µl of H_2_O and stored at −20 °C. Primers are listed in Table S4.

### Statistical analyses

All values were presented as means ± s.e.m. of three independent biological replicate. Two-tailed *t*-test was used for RT-qPCR, ChIP, proliferation, invasion and soft agar experiments. For frequency analysis in contingency tables, statistical analyses of associations between variables were performed by the Fisher’s exact test and for continuous variables the non-parametric Mann–Whitney *U* test. A *P* value < 0.05 was considered significant. SPSS software was used to perform all survival analyses and generate the log-rank *P* values. No statistical method was used to select sample size.

## Electronic supplementary material

supplementary Material

supplementary tables

supplementary Figure 1

supplementary Figure 2

supplementary Figure 3

supplementary Figure 4

supplementary Figure 5
