# Supplementary material for: Oncogenic activity of poly (ADP-ribose) glycohydrolase
Source: Oncogene. 2018 Nov 20;38(12):2177–91. doi: 10.1038/s41388-018-0568-6 (PMC6484711; doi:10.1038/s41388-018-0568-6)
Supplement: Supplementary file 1 — supplementary Material [file 41388_2018_568_MOESM1_ESM.docx]

**Supplementary figure legends:**

**Figure S1: PARG expression in cancer and its impact on breast cancer subtypes survival.** (a) Bar graph showing the percent of tumor samples with elevated PARG mRNA expression in various cancers using TCGA RNAseq data. (b) PARG antibody validation for IHC using embedded cell pellets from PARG-negative cell line (HMLE) and PARG-positive cell line (MDA-MB-231) with two dilutions of the antibody. (c) Breast cancer samples were classified according to molecular subtypes and survival was assessed based on PARG mRNA levels using TCGA RNA-seq data (PARG expression was separated by the median value). Basal-like (c), Luminal A (d) and Luminal B (e) Kaplan Meier plot was generated using PARG mRNA level (High or Low, split by median) in HER2-enriched (f) or HER2 negative (g) breast cancer. The P value associated with the Kaplan-Meier plot represents log rank test. (h) Western blot probing for PARG in a panel of breast cancer cell lines.

**Figure S2: Modulation of PARG level affects cellular transformation and tumor growth.** (a) Western blot against PARG and Actin were carried out with proteins extracts from HMLEN infected with Ctl vectors, PARGwt or PARGmut. (b) Western blot against FLAG and Actin were carried out with proteins extracts from HMLE infected with Ctl vectors, PARGwt or PARGmut. Western blot against PAR and actin were carried out following treatment of HMLE cells with PARP inhibitor MK-4827 for 24 hours (c) or doxorubicin, 30 minutes (d). (e) Proliferation curves of HMLE-Ctl, HMLE-PARGwt and HMLE-PARGmut cell lines. (f) PARG and actin western blot in HMLE-PARGwt infected with control shRNA or two different quantities of shRNA targeting PARG. (g) and (h) Bar graph representing the relative number of invading cells compare to HMLE-PARGwt infected with shRNA control (CT). Error bars represent mean ± s.e.m. Representative photos for each cell line are shown. (i) Western blot against PARG, Snail and actin were carried out in MCF10A TP53^-/-^ RB1^-/-^ infected with control vector (Ctl) or PARGwt. (j) and (k) Invasion assay using MCF10A TP53^-/-^ RB1^-/-^ Ctl and PARGwt. Bar graph represent mean ± s.e.m. Representative pictures for each cell lines are shown. (l) Soft agar colony forming assays using HMLE, HMLEN-Ctl and HMLEN-PARGwt cell lines. Bar graph representing percent of colonies on soft agar relative to HMLE cells. Error bars represent mean ± s.e.m. (m) Soft agar colony formation assays with HMLE, HMLEN-Ctl and HMLEN-PARGwt cell lines. Representative pictures for each cell lines are shown.

**Figure S3: PARG depletion reduces MDA-MB-231 aggressiveness.** (a) MDA-MB-231 knockdown cells probed for PARG. (b) Proliferation, (c) soft agar, (d-e) invasion and (f-g) migration assays in MDA-MD-231 control and PARG knockdown. Error bars represent mean ± s.e.m of three independent experiments. P values were calculated by two-tailed t-test. *P < 0.05, **P < 0.01, ***P < 0.001. (h) Tumor volume of NOD/SCID mice injected with 0.5x106 MDA-MD-231-M2 shCT or shPARG#05 cells.

**Figure S4: PARG depletion affects cellular transformation phenotypes in murine 66cl4 cell line.** (a) Western blotting against PARG and Actin using 66cl4 cell extract following PARG knockdown. (b) Wound healing experiment to test the migration potential of 66cl4 cells after PARG knockdown. Error bars represent mean ± s.e.m of three independent experiments. P values were calculated by two-tailed t-test. ***P < 0.001. (c-d) Invasion capacity of 66cl4 was tested following PARG depletion. Percent of cells invading through matrigel relative to 66cl4-shCT. Error bars represent mean ± s.e.m of three independent experiments. P values were calculated by two-tailed t-test. *P < 0.05.

**Figure S5: Phosphorylation of SMAD2/3 is not affected by PARG depletion.** (a-b) Western blot of PARG, phospho-SMAD2 (S465/467), phospho-SMAD3 (S423/425) and Nucleolin expression from MDA-MB-231-M2 shCT or shPARG cell extract following TGFβ (2.5ng/ml) treatment for 30min and 120min. (c) Western blot of Vimentin following TGFβ treatment in HMLE-Ctl and HMLE-PARGwt. (d) ChIP of SMAD3 at Vimentin following TGFβ treatment for 2h in HMLE-Ctl and HMLE-PARGwt. Error bars represent mean ± s.e.m of three independent experiments. P values were calculated by two-tailed t-test. *P < 0.05. (e) ChIP of SMAD3 at Vimentin following TGFβ treatment for 2h in HMLE-Ctl and HMLE-PARGmut. Error bars represent mean ± s.e.m. (f) Proliferation assay in HMLE-Ctl and HMLE-PARGwt following SMAD2 and SMAD3 knockdown after 4 days. Error bars represent mean ± s.e.m. (g) Western blot of SMAD2/3 and nucleoline were carried out in HMLE-PARGwt cells infected with shRNA control or shRNA targeting SMAD3. (h) Invasion assay were carried out in HMLE-PARGwt infected with shCT or shSMAD3-B. Error bars represent mean ± s.e.m of three independent experiments. P values were calculated by two-tailed t-test. ***P < 0.001.
