# Supplementary material for: Oncogenic activity of poly (ADP-ribose) glycohydrolase
Source: Oncogene. 2018 Nov 20;38(12):2177–91. doi: 10.1038/s41388-018-0568-6 (PMC6484711; doi:10.1038/s41388-018-0568-6)
Supplement: Supplementary file 2 — supplementary tables [file 41388_2018_568_MOESM2_ESM.docx]

**Supplementary tables**

Table S1: shRNA sequences

| Name | Cat # | Sequence |
| --- | --- | --- |
| PARG#05 | \| TRCN0000051305 \| \| --- \| | CCGGGCTGAGCGAGATGTGGTTTATCTCGAGATAAACCACATCTCGCTCAGCTTTTTG |
| PARG#06 | TRCN0000051306 | CCGGCGATTGCATGTCACTTACGAACTCGAGTTCGTAAGTGACATGCAATCGTTTTTG |
| SMAD3-A | TRCN0000330127 | CCGGGAGCCTGGTCAAGAAACTCAACTCGAGTTGAGTTTCTTGACCAGGCTCTTTTTG |
| SMAD3-B | TRCN0000330128 | CCGGTGAGCAGAACAGGTAGTATTACTCGAGTAATACTACCTGTTCTGCTCATTTTTG |
| SMAD2-A | TRCN0000010477 | CCGGCAAGTACTCCTTGCTGGATTGCTCGAGCAATCCAGCAAGGAGTACTTGTTTTTG |
| SMAD2-B | TRCN0000010478 | CCGGCATGATCCAGTATCACAGTATCTCGAGATACTGTGATACTGGATCATGTTTTTG |
| Parg#1 | TRCN0000126562 | CCGGGCAGTTTCTTACACCTATAAACTCGAGTTTATAGGTGTAAGAAACTGCTTTTTG |
| Parg#2 |  | CCGGAGTTCTGTGCAGAAAGATAACCTCGAGGTTATCTTTCTGCACAGAACTTTTTTG |

Table S2: Antibodies list.

| **Name** | **Company** | **#Cat** | **Usage** |
| --- | --- | --- | --- |
| **Western Blot** | | | |
| PARG (D8B10) | Millipore | MAB561 | 1/1000 |
| Actin | Sigma | A5316 | 1/5000 |
| VIM | BD biosciences | #550513 | 1/2500 |
| Nucleolin (C23) | SantaCruz | Sc-8031 | 1/2500 |
| PAR | Millipore | MABC547 | 1/1000 |
| SNAIL | Cell Signalling | 3895S | 1/1000 |
| SMAD4 | SantaCruz | Sc-7966 | 1/1000 |
| SMAD2/3 (D7G7) | Cell Signalling | 8685S | 1/1000 |
| P-SMAD2 (S465/467) | Cell Signalling | 3108S | 1/1000 |
| P-SMAD3 (S423/425) | Cell Signalling | 9520S | 1/1000 |
| CTCF | BD biosciences | #612149 | 1/1000 |
| GAPDH | Origene | TA802519 | 1/2500 |
| MYC (9E10) | SantaCruz | Sc-40 | 1/2500 |
| Goat anti-mouse | KPL | #474-1806 | 1/5000 |
| Goat anti-Rabbit | KPL | #474-1516 | 1/5000 |
| **ChIP** | | | |
| SMAD2 | Abcam | Ab71109 | 4 ug/IP |
| SMAD3 | Abcam | Ab208182 | 4 ug/IP |
| **IHC** | | | |
| PARG | Novus | NBP1-89450 | 1:25 |
| LYVE1 | Abcam | Ab14917 | 1:100 |
| **IF** | | | |
| VIM | BD biosciences | #550513 | 1:500 |
| Phalloidin Alexa 568 | Invitrogen | A12380 | 1:500 |
| SMAD2/3 | Cell Signalling | 8685S | 1:500 |
| Alexa 488 goat anti-mouse | Invitrogen | A11029 | 1:1000 |

Table S3: RT-qPCR primer sequences.

| Name | Sequence |
| --- | --- |
| 36B4 | 5-CGACCTGGAAGTCCAACTAC-3 |
|  | 5-ATCTGCTGCATCTGCTTG-3 |
| PARG | 5-GAGGTGCTGGATCACAATGA-3 |
|  | 5-TCTCAGGCACAAACTGATCG-3 |
| VIM | 5-GAGGAAGCCGAAAACACCCT-3 |
|  | 5-TTGCGTTCAAGGTCAAGACG-3 |
| FN1 | 5-AGGATGACAAGGAAAGTGTCCC-3 |
|  | 5-CCTCAGGCCGATGCTTGAAT-3 |

Table S4: ChIP primer sequences.

| Name | Sequence |
| --- | --- |
| VIM +400 | 5-CAGGACTCGGTGGACTTCTC-3 |
|  | 5-GTCGATGTAGTTGGCGAAGC-3 |
| VIM -1200 | 5-CCGCCAAAGATTCTGTCATT-3 |
|  | 5-GTGGTTTTTACCCTGGTGGA-3 |
| VIM +5500 | 5-GAGTGACTTTCGGGGAATGA-3 |
|  | 5-CTCCTGGATTTCCTGCAAAA-3 |
| FN1 TSS | 5-CCTTTGCGGTCATCAAACTT-3 |
|  | 5-CTTCGCTTCACACAAGTCCA-3 |
| FN1 +20kb | 5-CGGAGCAAACCAGACATACA-3 |
|  | 5-AGCAAGATCCCACTGAGCAT-3 |
| Negative region | 5-CTCCTTCATTTGAGGGGAAA-3 |
|  | 5-TCACTGGTGGACTTGGTTGA-3 |
