# Supplementary figures and images for: Oncogenic activity of poly (ADP-ribose) glycohydrolase
Source: Oncogene. 2018 Nov 20;38(12):2177–91. doi: 10.1038/s41388-018-0568-6 (PMC6484711; doi:10.1038/s41388-018-0568-6)

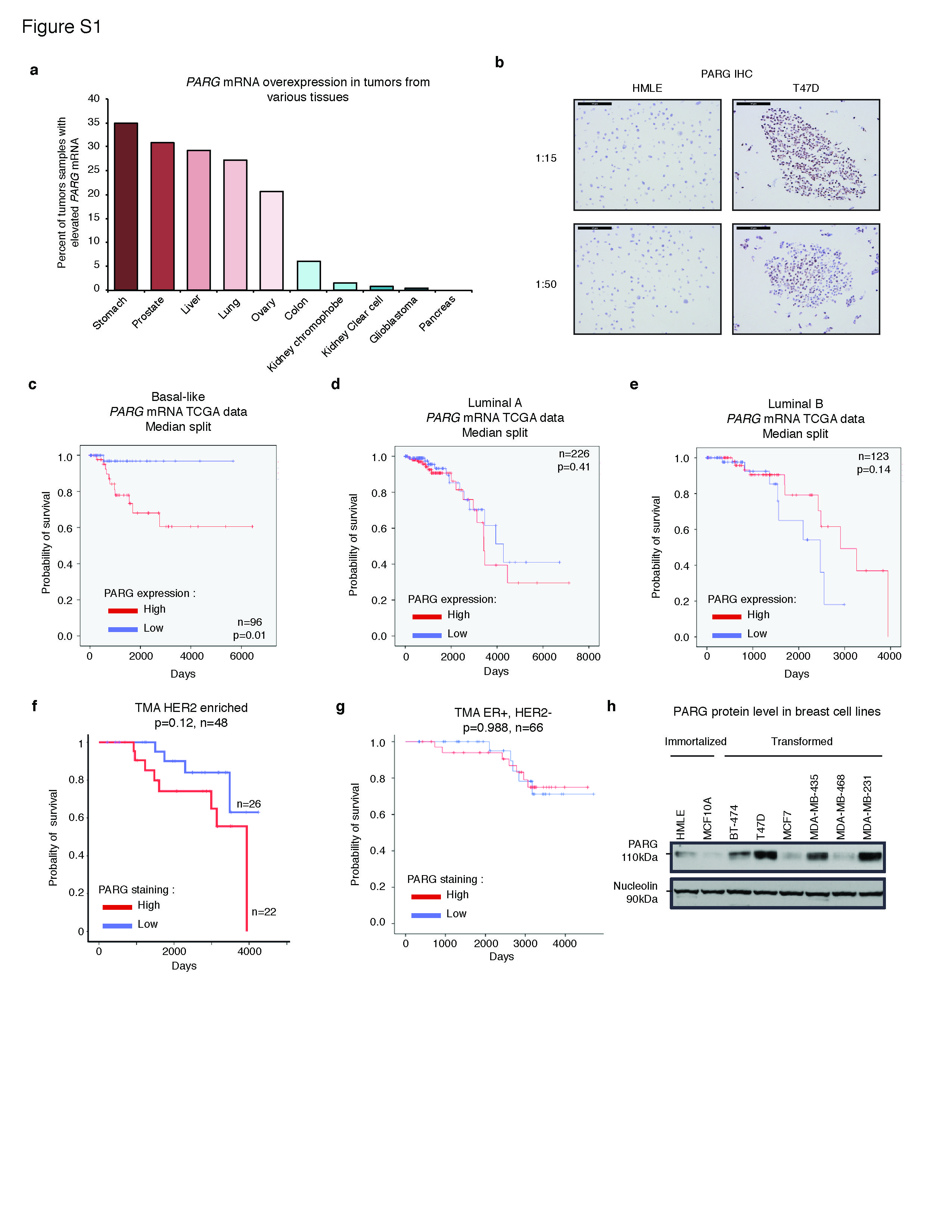

Supplement: Supplementary file 3 — supplementary Figure 1 [file 41388_2018_568_MOESM3_ESM.jpg]

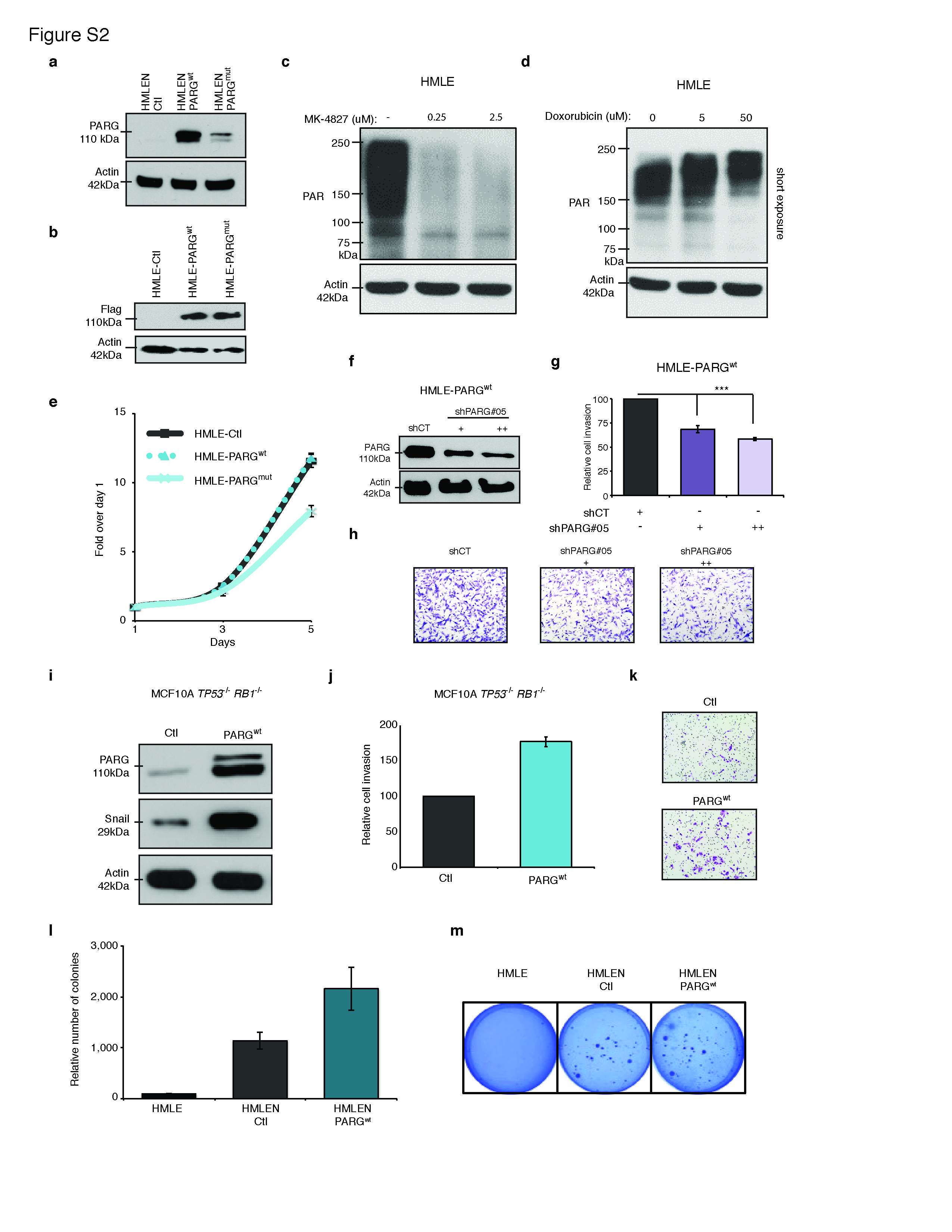

Supplement: Supplementary file 4 — supplementary Figure 2 [file 41388_2018_568_MOESM4_ESM.jpg]

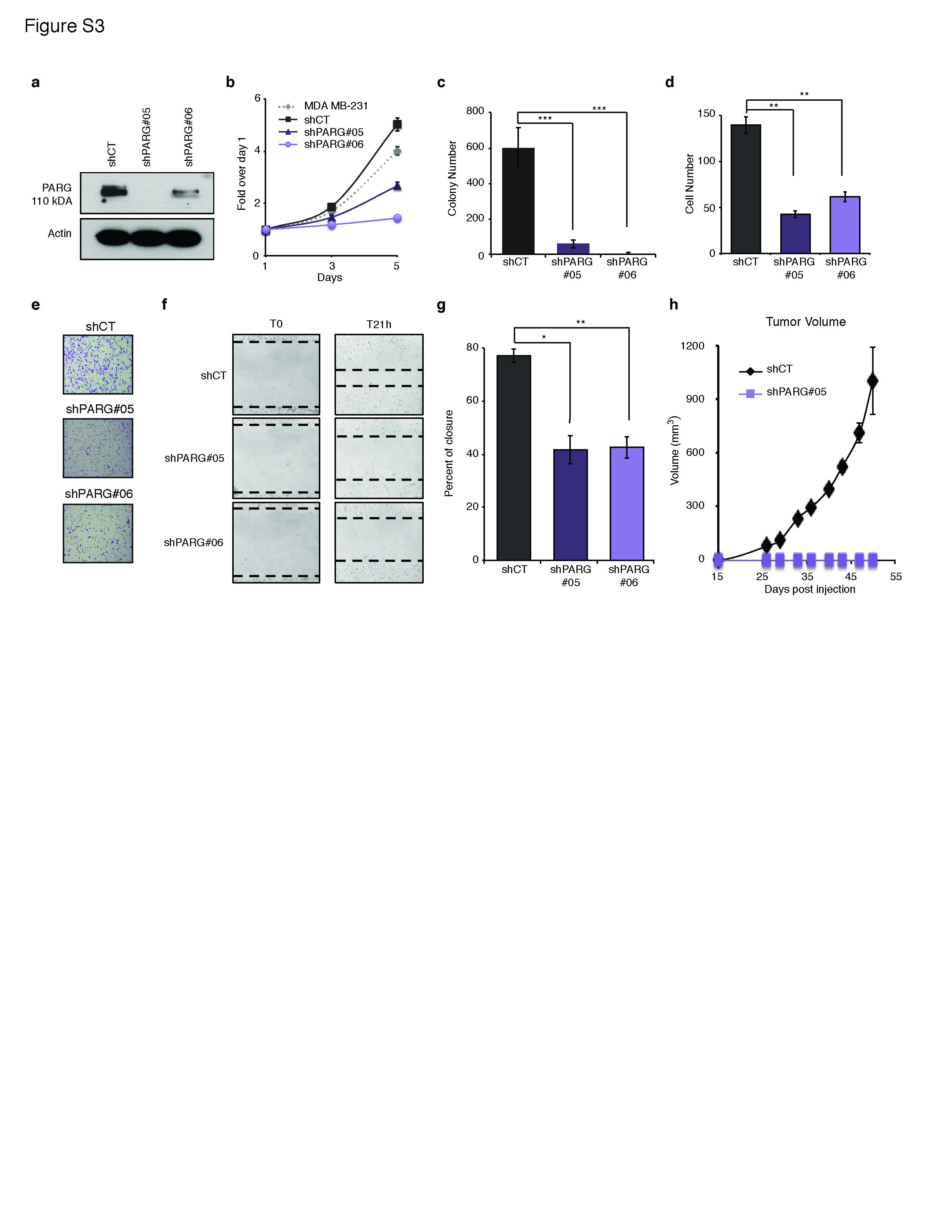

Supplement: Supplementary file 5 — supplementary Figure 3 [file 41388_2018_568_MOESM5_ESM.jpg]

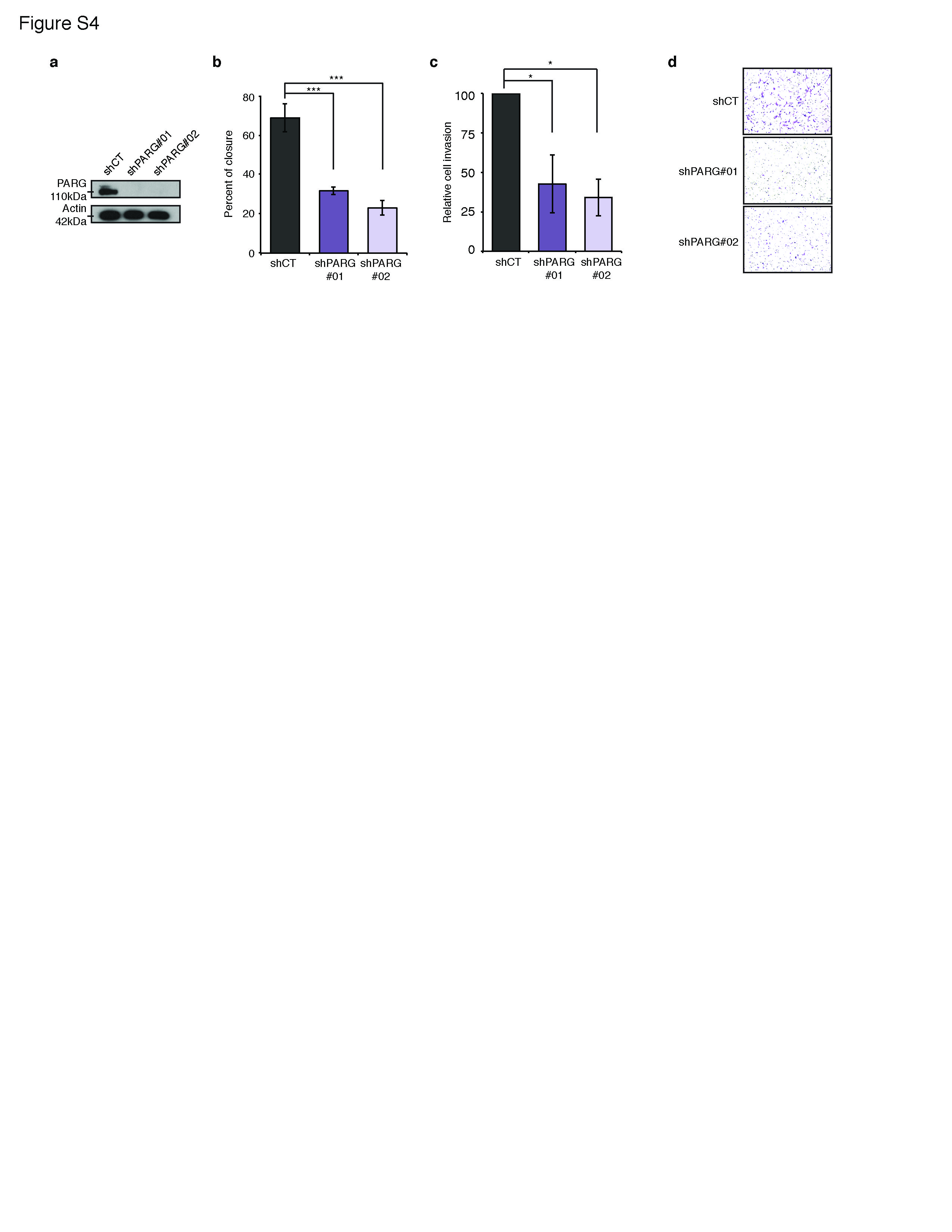

Supplement: Supplementary file 6 — supplementary Figure 4 [file 41388_2018_568_MOESM6_ESM.jpg]

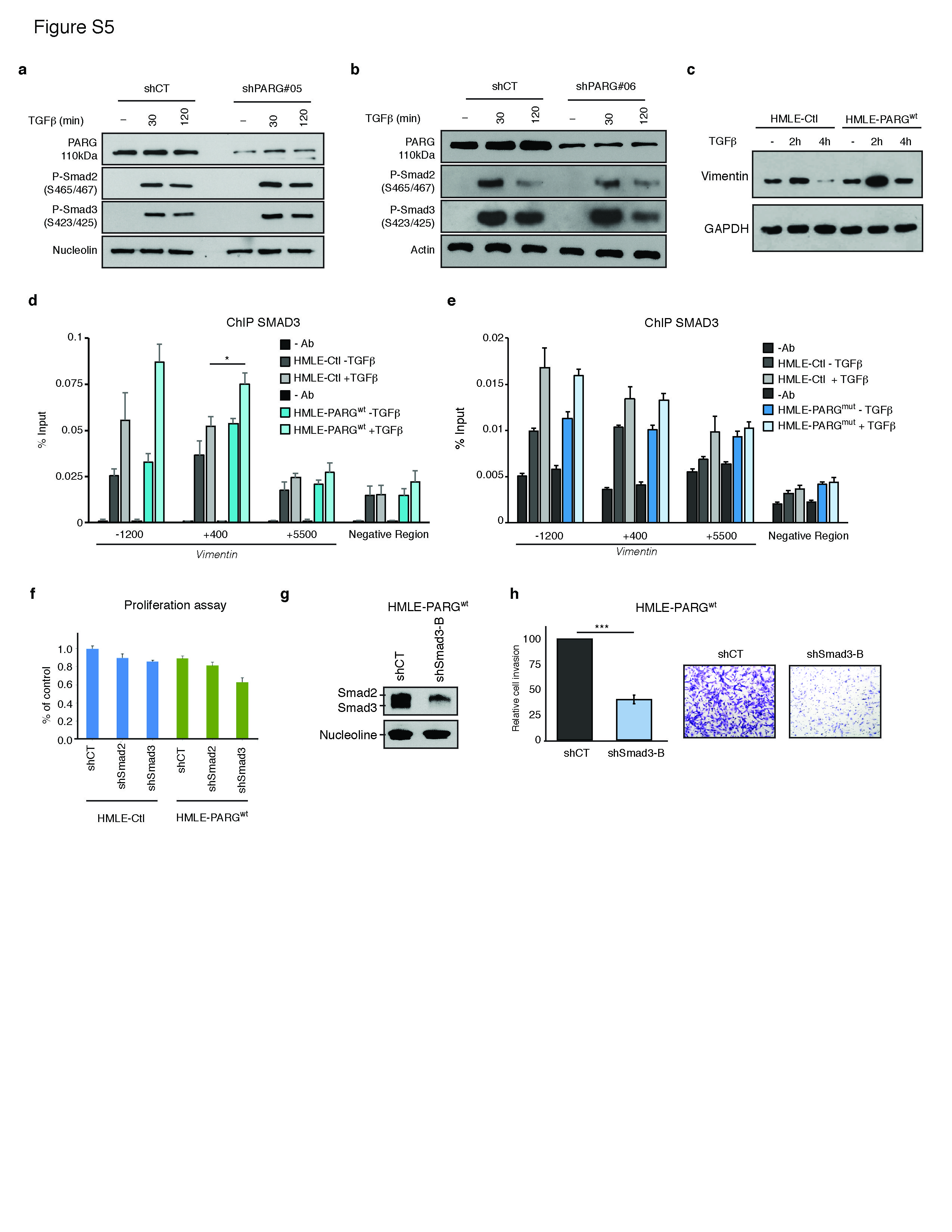

Supplement: Supplementary file 7 — supplementary Figure 5 [file 41388_2018_568_MOESM7_ESM.jpg]
